# What are patterns of rise and decline?

**DOI:** 10.1098/rsos.230052

**Published:** 2023-11-15

**Authors:** Aura Raulo, Alexis Rojas, Björn Kröger, Antti Laaksonen, Carlos Lamuela Orta, Silva Nurmio, Mirva Peltoniemi, Leo Lahti, Indrė Žliobaitė

**Affiliations:** ^1^ Department of Computing, University of Turku, Turku, Finland; ^2^ Department of Biology, University of Oxford, Oxford OX1 3SZ, UK; ^3^ Department of Computer Science, University of Helsinki, Helsinki, Finland; ^4^ Finnish Museum of Natural History, University of Helsinki, Helsinki, Finland; ^5^ Mobility Research Group, VTT Technical Research Centre of Finland, Espoo, Uusimaa, Finland; ^6^ Department of Languages, University of Helsinki, Helsinki, Finland; ^7^ Department of Industrial Engineering and Management, Tampere University, 33014 Tampere, Finland; ^8^ Department of Geosciences and Geography, University of Helsinki, Helsinki, Finland

**Keywords:** rise and decline, hat pattern, evolution, unimodality, emergence, system dynamics

## Abstract

The notions of *change*, such as birth, death, growth, evolution and longevity, extend across reality, including biological, cultural and societal phenomena. Patterns of change describe how success and composition of every entity, from species to societies, vary across time. Languages develop into new languages, music and fashion continuously evolve, economies rise and decline, ecological and societal crises come and go. A common way to perceive and analyse change processes is through patterns of rise and decline, the ubiquitous, often distinctively unimodal trajectories describing life histories of various entities. These patterns come in different shapes and are measured according to varying definitions. Depending on how they are measured, patterns of rise and decline can reveal, emphasize, mask or obscure important dynamics in natural and cultural phenomena. Importantly, the variations of how dynamics are measured can be vast, making it impossible to directly compare patterns of rise and decline across fields of science. Standardized analysis of these patterns has the potential to uncover important but overlooked commonalities across natural phenomena and potentially help us catch the onset of dramatic shifts in entities' state, from catastrophic crashes in success to gradual emergence of new entities. We provide a framework for standardized recognizing, characterizing and comparing patterns of change by combining understanding of dynamics across fields of science. Our toolkit aims at enhancing understanding of the most general tendencies of change, through two complementary perspectives: dynamics of emergence and dynamics of success. We gather comparable cases and data from different research fields and summarize open research questions that can help us understand the universal principles, perception-biases and field-specific tendencies in patterns of rise and decline of entities in nature.

## 1. Introduction

The world as we know it is changing at unprecedented rates. Financial crises, epidemics, ecological crises and military conflicts arise suddenly and change ways of living. At the same time, new cultures, innovations and industries emerge and gain success at an increasing pace. The world is rarely at equilibrium [1]—by the time we learn how the world is, it has already changed. Language inventories provide evidence that languages change in terms of number of users and structural features [2], computational analysis of music shows how Western classical music gradually evolved from Baroque to modern music[3]archaeological records show that civilizations emerge, prosper and disappear [4], and entrepreneurship scholars have described how firms emerge, grow and eventually decline [5]. Entities of life and culture may emerge as new combinations of features rise and decline in success and eventually disappear through either abrupt crashes or protracted attritional phases.

Patterns of change occurring across different contexts may be remarkably similar. For instance, particular fluctuations in a system's state, called ‘early-warning signals' [6] have been shown to predict sudden shifts and collapses in contexts ranging from ecological communities [7] and global climate [8] to housing markets [9] and even mental health [10]. Similarly, hat-shaped unimodal success curves that describe the birth, rise, peak, decline and disappearance of an entity across time have been identified in biological, cultural and economic systems [11,12]. Success curves of both biological species [13] and scientific fields [14] tend to show a similar right-tailed curve, a rapid rise followed by a gradual decline over time.

Although patterns of rise and decline have interested scholars for centuries and have been described across several entities in nature, including society, efforts to compare these patterns of change across disciplines are scarce. Few lines of interdisciplinary research on universal dynamics exist, including the theory of *complex systems*, which makes use of chaos theory and network theory in modelling physical, biological and cultural systems as networks of interacting components[15]and *Holling's life cycle theory,* which describes social and ecological systems both as sets of differently paced processes hierarchically nested inside each other [16]. These theories have an emphasis on modelling mechanisms driving certain dynamics observed, or expected to happen, in social and ecological entities. However, even here comparing patterns of change across different systems is hard to do, as the focus and means of measuring any meaningful dynamics varies greatly between research fields and data. Building a standardized framework for recognizing and quantifying patterns of rise and decline, particularly patterns of *emergence,* subsequent patterns of *rise and decline in success,* as well as *disappearance* in a way that is comparable across different contexts and time scales is the primary goal of our present study. Our aim is to provide methods for recognizing and comparing dynamics across data and research fields, to foster more data-driven research on the universality of patterns of change. We leave analysis of processes driving these patterns of change for future studies. Alongside this framework, we discuss to what extent our perception and analysis over patterns of change may be influenced by historical and cultural traditions of thought.

Let us consider natural *entities*, such as species within ecosystems, languages within communities, or industries within economies. Within these realms, entities live in an ever-changing environment and, to a varying extent, compete for the same resources. The changing environment and competition favour processes that create new variations of the entity. They keep all the entities ‘on the move’: whenever change, even random change, is possible, every entity must change or perish, because staying the same would mean becoming misfit and being eventually out-competed by the changing others (a principle called the Red Queen's hypothesis [17]). Driven by such forces of competition, we see new entities come into existence and old ones become extinct. The emergence, growth, decline and disappearance of these entities can be measured in multiple ways. We can trace the trajectory of a biological species or a language over time by looking at the dynamical process by which they emerge (e.g. how a set of linguistic elements changes enough to be considered a new language or dialect; how a set of traits or gene variants become to constitute a species) or by measuring their success, i.e. abundance of individuals it comprises, or the geographical area that the entity covers at different times. In both cases, the rise and decline may be orchestrated by various different processes. For example, an entity may emerge as a result of reorganization of some set of features (e.g. a new species emerging through gradual optimization over combinations of gene variants) or as a result of instantaneous merging of two pre-existing sets of features (e.g. a new species emerging through hybridization of two species). After emergence, an entity may expand by multiplying its units (e.g. reproduction in biological species, learning a language from one's parents) or through an external influx (e.g. a language becoming more commonly spoken due to immigration).

**Figure 1.**
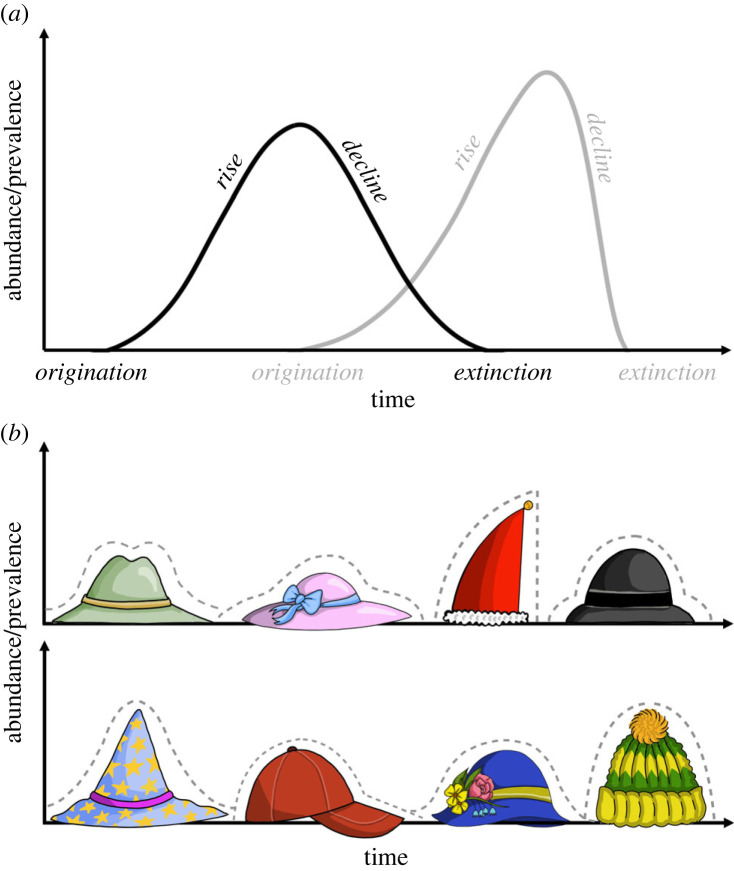
General trajectories and variations in shape of patterns of rise and decline. (*a*) A conceptual pattern describing the trajectory of rise and decline in success, such as abundance or prevalence, across the history of entities within a natural system. (*b*) Examples of different shapes of ‘hat patterns’ as illustrated by different types of hats. For example, hat patterns can be steep or flat, symmetrical or skewed, or more or less unimodal.

Figure 1*a* illustrates the simplest conceptualization of patterns of change over time. This curve depicts the rise and decline in some measure of success, such as abundance over the lifespan of an entity. Over time, the success of an entity rises, peaks and starts declining until the entity becomes rare and eventually ceases to exist while being replaced by another entity. Although there can be multiple peaks of success (e.g. relative abundance of omnivorous mammal species across their history [18]; or revival of a language [19]), the simplest unimodal pattern is remarkably common across natural entities [11,12,18]. We refer to this pattern as a hat-like success curve or a ‘hat pattern’ [13]. The architecture of the hat patterns varies (figure 1*b*). For instance, rise and decline in success can be symmetric or asymmetric, more or less unimodal, following trajectories of different shapes and smoothness or showing varying frequency of smaller scale fluctuations. A full trajectory pattern is only seen in retrospect, at the end of the lifetime of an entity.

Patterns of change are largely studied in isolation across research fields. Yet, standardized methods are essential for scientific advances. With more standardized descriptions of change processes, we can lend understanding from one context to another to provide tools to compare, anticipate and manage change, from rapid shifts in stock markets to slow progress of language change. While the thresholds and triggers of change operate at different scales, the general tendencies can be transferable across contexts. For example, studies on the survival of languages [2] as well as animal species [20,21] have identified the minimum viable population size (minimum success) for a declining language or species, below which they are unlikely to recover. With a standardized framework these methods could be generalized to identify critical markers of extinction risk for other entities, such as industries or cultural practices. Standardized study of change processes can also help us better understand phenomena such as *emergenc*e, *extinction* and *ageing*—when, why and how entities tend to appear or disappear. Do species, economies, languages and cultures age in a comparable way, could their decline ever be predictable from their state and how can we separate when the extinction of an entity is a result of its gradual evolution into another entity, a phenomenon sometimes called pseudoextinction [22], compared to a terminal extinction of a lineage?

Our study focuses on examples from six fields of inquiry: macroevolutionary studies, microbial ecology, linguistics, musicology, industry evolution studies and human mobility studies. These fields study processes that happen at different timescales, they consider phenomena that are complex, noisy and have hierarchical structure, i.e. ‘complex’ as in their behaviour/dynamics cannot be described by mechanical mathematical models alone, as they exhibit emergent behaviours, ‘noisy’ as in they are studied with data containing uncertainty and ‘have hierarchical structure’ in that we can describe the structure of studied entities as compositions of lower-level units. First, we review the varying discipline-specific traditions in describing patterns of change. Then, we suggest a way of standardizing this variation by identifying comparable entities across the different research fields, and suggest comparable definitions for their units and measures of emergence and success across time. Finally, we gather a toolbox of simple methods for visualization and quantitative analysis of these patterns, which enhance our ability to compare dynamics across different contexts.

## 2. A historical account on recognizing and analysing patterns of rise and decline

### 2.1. The notion of rise and decline in historiographical research of civilizations

**Figure 2.**
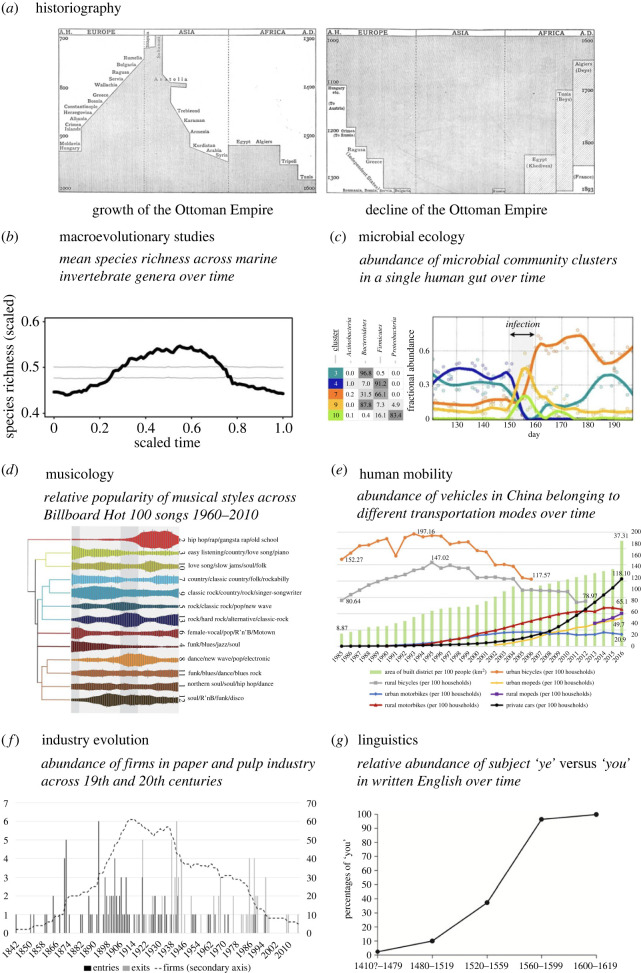
Examples of patterns of rise and decline from studies in the different fields of inquiry. (*a*) Series of histograms depicting the rise and decline in the geographical area of the Ottoman Empire across centuries [24]. (*b*) Mean trajectory (black line) of species richness across genera of marine invertebrates [25]. (*c*) Fractional abundance of different microbial community (co-occurrence) clusters, each corresponding to one dominant bacterial phylum, over time in one single human's gut [26]. (*d*) Relative popularity (frequency across songs on Billboard Hot 100 list) of music genres across five decades [27]. (*e*) Abundances of ownership of vehicles belonging to different transport mode categories in China across time [28]. (*f*) Abundance of firms in paper and pulp industry across nineteenth and twentieth centuries [29]. (*g*) Replacement of subject ‘ye’ by subject ‘you’ over time in written English [30].

One of the first academic notions of rise and decline of any entity is the idea of rise and decline of civilizations, a common theme across the study of historiography. Human civilizations were perceived to have an anthropomorphized lifespan—to be born, to grow, to age and eventually die—by Herodotus already before 400 BC, writing about the history of the Persian empire. In modern times, Herodotus' narrative was echoed by Edward Gibbon in his 1776 book *The History of the Decline and Fall of The Roman Empire* [23]. In Europe, this was a particularly influential piece of work, rooting the idea of rising and falling civilizations and inspiring studies of cultures as living organisms with a waxing and waning lifespan into the canon of Western historiography. For instance, Lane-Poole [24] summarizes ‘the growth and decline of the Ottoman Empire’ in a series of histograms depicting the hat-like pattern in the geographical area covered by the empire across the centuries (figure 2*a*). Likewise, in *A* *Study of History*, Toynbee describes civilizations as inherently waxing and waning entities [31]. Similarly, inspired by Goethe's idea of cultures as living organisms, Spengler [32] suggested that civilizations too have a life cycle of an organism, that is they are born, grow, mature, age and die in a predictable manner. In his book *The* *Rise and Fall of Civilization*, Clough takes this notion as granted in his exploration into what might be the reasons behind such universal patterns [33]. The narratives of deterministic inevitability of civilizational decline were abundant in the justifications of European imperialism as well as the eugenic programme [34], and subsequently heavily criticized by modern historians, perhaps most influentially Edward Said [35], emphasizing the Western bias in such narratives.

### 2.2. The notion of rise and decline in biology

Paralleling the temporal narratives in historiography, patterns of rise and decline have been long recognized in sub-fields of biology, ranging from microbial ecology (e.g. epidemic waves caused by viral strains) to developmental biology (e.g. molecular ‘signalling waves’ during embryonic development) and perhaps most notably macroevolutionary studies (e.g. success curves of species across their life span). In a pioneering assessment of the British fossil record, Phillips [36] observed that ‘prevalent fauna begins at a minimum abundance, rises to a maximum, and dies away to a final minimum, to be followed by another system having similar phases’. In his origin of species by natural selection, Darwin [37] also described a hat pattern, noting that ‘large genera (those with a large number of species) have often come to their maxima, declined, and disappeared’. Nearly a century later Simpson [38] laid foundations for analysing macroevolutionary processes quantitatively, introducing the concept of tempo of evolution. Quantitative analysis of such patterns has been conducted on the fossil record of mammals [18], marine invertebrates [25,39] (figure 2*b*), and microorganisms [40]. Based on this, the abundance, occupancy (prevalence) and geographical range histories of individual species and genera retrieved from the fossil record exhibit a variety of shapes, the simplest and most generic trajectory pattern often exhibiting a single prominent peak (unimodal pattern). Consequently, the term ‘hat pattern’ was originally composed in the field of macroevolutionary studies [13].

The idea of change of entities, i.e. how languages, industries, cultures or species vary from time to time, is inherently related to notions of evolution. Palaeontology and evolutionary biology are not the only research fields that refer to *evolution* when assessing the emergence or rise and decline of entities. The varying semantics of ‘evolution’ and other terms for change across different fields of inquiry are summarized in electronic supplementary material, appendix S1. However, across biology and other research fields, there are also patterns of rise and decline not traditionally attributed to ‘evolutionary change’. For instance, patterns of rise and decline similar to those observed in the fossil record, have been observed in the *ecological succession* of species, such as the change in the composition of plant species across time in a maturing forest. Ecological succession is not considered an example of evolutionary change but rather ecological change in biology. The theory of ecological succession was formalized by Cowles [41], who noted that change in plant communities was driven by a sequence of success curves of individual plant species. This is reminiscent of ecological dynamics in microbial communities [42], whereby bacterial lineages are frequently observed to go through rapid ecological successions. This phenomenon has been historically referred to as ‘blooms’ in free-living microbial communities (from ‘water-blooms’ first mentioned by Marsh in 1900 [43]), ‘invasions’ in the context of internal gut microbiomes (e.g. [44]) or ‘waves’ in context of epidemics. The wave metaphor was popularized by Arthur Ransome in 1882, when he, describing the spread of disease, wrote ‘The course of an epidemic disease through a country may aptly be compared to a wave, gradually rising and then falling’ [45]. In all of these cases, microbial succession dynamics provide examples of hat patterns, with typically a unimodal peak followed by an inevitable decline and extinction of a particular microbial lineage from a particular community (figure 2*c*).

Ecological and evolutionary dynamics are not independent of each other: natural selection that gives rise to novel biological entities depend on the ecological community dynamics in which the organisms live. Our exemplified cases of macroevolutionary patterns and ecological succession events differ remarkably in temporal scale, but evolutionary and ecological change can also happen intertwined in similar temporal scales [46]. This is the realm of research in ‘eco-evolutionary feed-back loops', such as the rapid and repeated wave-like evolution of traits in communities of species living under fluctuating natural selection caused by fluctuating ecological situations [47–49]. For example, predator–prey experiments have demonstrated the repeated evolution, rise and decline of certain predator defence traits in planktonic algae: when the defence mechanism evolves, it initially rises in abundance but soon becomes obsolete as predators diminish, consequently declining as it becomes a costly but useless trait, followed by an increase in predator population that selects for the re-evolution of the defence mechanism [47]. Similar patterns of rapid evolutionary success curves have been also documented in traits under negative frequency-dependent sexual selection. For example, colour patterns in male guppy fish exhibit wave-like patterns of frequency, as the males are selected by females based on rarity of their colour—locally new colours tend to become increasingly common until their commonness makes them uninteresting to the females, after which they decline in frequency [48].

### 2.3. The notion of rise and decline in cultural and societal phenomena

The perception of change in human activity as a sequence of wave-like rise and decline patterns of some loosely defined socio-cultural entities is common across the social sciences, such as in the study of fashion or music styles. A pioneering example comes from A. Kroeber, who in 1919 analysed changes of women's fashion in Europe across the nineteenth century, suggesting that the success curves of different styles in arts, literature and fashion reflect the same universal patterns observed in the fates of civilizations at large [50]. In his study ‘On the principle of order in civilization as exemplified by changes of fashion’ he writes: ‘Most social phenomena are expressible by nearly similar and presumably simple geometrical curves…each of these isolable movements (of art, theatre and literature) has been traced through a similar course of origin, growth, climax, decline, and either death or petrifaction, analogous to the life stories of organisms'. To date, patterns of rise and decline have been quantitatively identified in a number of studies over history of human subcultures, including modern fashion trends [51], musical styles [27] and movie genres [52]. For instance, the history of Western classical music can be divided into wave-like periods of predominant styles such as Baroque, Classical, Romantic [3]. Likewise in modern music, genres that dominate the industry are replaced by others over time (figure 2*d*) [27].

Paralleling cultural history, successive waves of rise and decline have also been recognized and analysed in other social science fields, such as studies of human infrastructure and economics. Human mobility studies have recognized the rise of different transportation modes as a hat pattern with a single ‘peak’ [53–56]. For example, a unimodal hat pattern has been documented in the popularity of traditional bicycles in China, where the decline phase coincided with the rise of electric bikes (figure 2*e*) [56], creating sequential waves similar to those in music history or in the ecological succession of plant communities. Similarly, patterns of rise and decline have emerged as a research topic in economics. For example, industries such as Finnish paper and pulp industry have gone through a hat-shaped success curve (figure 2*f*) [29]. Importantly, the dynamics of industries can be an independent process from any underlying cultural processes. This is the realm of ‘evolutionary economics', introduced by Nelson & Winter [57] as the study of variation and selection of *routines*. Routines, in economics, refer to ways of doing things, such as managing, recruiting or communicating within an organization, that remain relatively stable over time. Thereafter, industry life-cycle theory [58] and organizational ecology [59,60] have focused on studying long-term changes in industries. Here, the former sees organizations unable to make changes to adapt to environmental change, whereas the latter affords firms the capability of making strategic choices. Therefore, in organizational ecology, population-level change comes from old firms being replaced by new ones, whereas in industry life-cycle theory firms can also change what they do and how they do it.

While rise-and-decline narratives are common in many fields of social sciences, in historical linguistics rise and decline patterns are rare. However, they are implicitly assumed in the story of languages arising (by splitting off from an earlier ancestor) and becoming endangered or going extinct [61,62]. However, due to the difficulties of estimating speaker numbers (both in the present and in the past), such patterns tend not to be easy to quantify numerically, nor is this a major focus in the field. The temporal dynamics of emerging linguistic features can, however, be quantified more accurately and is often modelled by s-curves (sigmoid function) which can be viewed as the first half of a hat pattern. For instance, the rise of an innovation (e.g. sound change) is often modelled by lexical diffusion theory and characterized through an s-curve depicting the onset of a new feature, first progressing slowly, then accelerating in frequency before plateauing (figure 2*g*) [63–65].

## 3. Methods for analysing patterns of rise and decline

### 3.1. Characterizing success of an entity

As a starting point to analyse patterns of rise and decline, let us focus on the life history curve of an entity, such as a biological species, a language, a music genre or an industry across time (figure 1). The rise depicts the increase in success of the entity towards its peak, whereas the decline depicts the decrease in success towards its eventual disappearance. The horizontal axis denotes time, but the vertical axis (quantity of success) takes many forms.

The success of an entity can be any measure of the magnitude of its presence at a particular time, such as abundance (e.g. number of individuals belonging to a language community or a species), range (e.g. geographical range of species or cities, breadth of ecological niche of a species or range of contexts in which a language is used), value or market share (in firms or industries) or popularity (e.g. number of mentions of a fashion trend). When success is measured through the abundance of units, an entity can grow both by multiplying their units internally or by new units joining from outside. These processes are sometimes known as *vertical* or *horizontal transmission* in both biology and social sciences. For instance, a speaker community of a language can grow by increasing the number of people through reproduction or immigration and industries can grow by multiplying firms or by swallowing firms from other industries.

**Figure 3.**
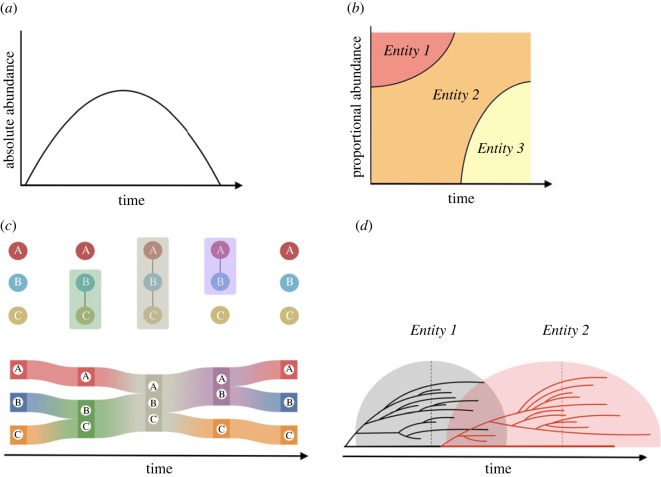
Alternative tools to visualize patterns of rise and decline. (*a*) A success curve and a spindle diagram, both describing a pattern of rise and decline of success, based on absolute abundance of units, e.g. rise and decline of number of individuals of a given species or chart-topping songs of a given musical genre across time (see e.g. fig. 3b in [25]; fig. 10 in [3]). (*b*) A Muller plot describing a pattern of rise and decline in success based on relative abundance of units, e.g. proportion of individuals belonging to each species or proportion of chart songs belonging to each musical genre across time (see e.g. fig. 2 in [66]). (*c*) Alluvial diagram [67] describing a pattern of emergence and dissolution of an entity through braiding together and unravelling of a specific combination of features, e.g. temporal clusters in covariation networks of gene variants among animal populations or harmonic and timbral elements among chart-topping songs across time (see e.g. fig. 4 in [68]). (*d*) Our suggestion of a tree plot describing the emergence and success dynamics of entities that are lineages descendant of each other, e.g. the emergence of a new microbial strain from a pre-existing strain and the success wave of the new strain as it multiplies (branches), reaches its peak branch number (dashed line) followed by a decline in branch number. Another variation of this is a spindle tree diagram, where in place of branch number, branch thickness is used to visualize success within a branch, such as number of units within a given lineage (see e.g. fig. 3 in [27]).

Quantifying the abundance of entities can be done in absolute or relative terms (figure 3*a*,*b*). Depending on the emphasis of analysis, we may simply count how the number of units (e.g. users of a given language) changes through time or we may quantify the relative share of the operating space occupied by the units of a given entity (e.g. the proportion of individuals speaking a certain language out of all the individuals in a community). The absolute count does not take into account potential constraints on resources and in principle permits perpetual growth of an entity. The relative measurement implies that if one entity rises, others should decline in relative terms. While most often entities compete for the same limited space or resources, new entities may also introduce new space or resources into the overall system. This is the case when an entity evolves an innovation (often termed as ‘key’ innovation) in using the resource in a way no other entity has done before, such as when a biological species evolves the ability to live on a diet previously not used by other species—in this case their success is not taking resources away from other species in the ecosystem. Similarly, when a language begins to be used in a context in which no language has been used before, such as in the dawn of Internet cultures, it is initially expanding to a territory without outcompeting resources from other entities.

One of the main challenges in analysing and comparing how entities rise and decline in success is the dynamic nature of entities' boundaries. As an example, delineating species from a continuous variation between sister lineages is not always straightforward. Species are made up of populations with an ever-shifting distribution of individual variation, but practical definitions of species are often tied to a limited number of individuals measured at a limited number of time points. However, to measure the success of an entity, it needs to have delineated boundaries, based on either intuition, tradition, expertise or quantitative methods that describe the emergence of an entity's content. For instance, in many cases biological species delineated via standard taxonomic procedures reproduce those entities recognized in the traditional knowledge of local communities [69].

### 3.2. Characterizing the emergence of an entity

New entities do not usually appear out of nowhere, but rather emerge as an accumulating combination of some pre-existing or newly evolved lower-level features. Thus, in addition to success dynamics, we can view the lifespan of an entity through patterns of *emergence and dissolution*. How did an entity become what it is? How did it cease to be what it was? These questions consider dynamics whereby the boundaries of the entity are not a prerequisite but rather the consequence of the analysis. In fact, analysis of emergence dynamics can help us identify entity boundaries (e.g. establishing when something has changed enough to be considered a new entity) to aid measuring its subsequent success dynamics across time.

Analysis of emergence dynamics sees the entity arising as a specific combination of some features (i.e. a language as a combination of linguistic elements or a species as a distribution of gene variants) and dissolving as this combination decouples in time. These features are not the same as the units we focused on as a measure of success. Following Gould [70], an entity can be composed of two types of sub-units: ‘interactors’ (e.g. individuals of a species or a language community) or ‘features’ (e.g. the gene variants that comprise a species, linguistic elements that make up a language system). ‘Interactor’ units are more redundant and interchangeable, whereas ‘features’ generally carry more irreplaceable functions as part of the entity. In other words, if one interactor, such as an individual, is removed, the identity of the species would not be greatly changed whereas changing a feature such as a characteristic trait or a gene variant can have great consequences on the identity of the species as a whole. Similarly, removing some individual users of a language often would have a minimal effect on the language as a whole, but removing some of the words or grammar can significantly affect the language as a whole.

Shifts in the abundance or composition of both types of units can in principle be used to analyse how an entity changes across time, but measuring the success of an entity is best done with interactor-like units. When a language declines because of a diminishing speaker or signer population, it loses its users but perhaps less so its features. This is directly related to the fact that interactor units are more redundant, i.e. they can be lost en masse with minimal effect on the identity of the entity. However, a drastic reduction in the number of interactors starts to have implications for the entity as a whole. This is because a certain number of interactors are needed to retain the feature variation required for an entity to properly function. For instance, when a species is critically endangered, even death of one individual can mean loss of a significant proportion of remaining genetic variation that constitutes that species. In contrast with success dynamics, emergence dynamics are best analysed from feature co-variation, such as co-occurrence of features across units. Here, we can use network analysis methods to evaluate the dynamics of emergence. In particular, community detection on network representations can be used to delineate entities from the underlying data (reviewed by Doreian *et al*. [71] and Coscia [72]). Different community detection methods exist for identifying and delineating clusters of underlying features, for example through identifying areas of high edge density [73] or topological similarity [74] among nodes in a feature covariation network or through exploring community structure with random walks across a higher order network of features and units [75]. These methods can capture dynamics of covariation clusters yielding insights of how new entities emerge. For instance, when people follow different fashion styles or music genres at different times, we can see the rise of a given style emerge first as a gradual rise of a new combination of features that occur increasingly together (e.g. a novel combination of musical elements that becomes a new genre). Subsequently, we may then follow the rise in the success of this entity through proportional increase in the popularity of this combination (style/genre) in relation to others present among the same people.

In principle, we can analyse success dynamics of entities without paying attention to how they emerged. However, parallel analysis of dynamics in an entity's success and content can give us important insights. Firstly, it gives us the ability to perceive patterns of descendance and delineate different types of disappearance among entities. When we focus solely on success dynamics, we miss the insights over which entities go extinct leaving no descendants and which entities cease to exist by evolving into new entities. For example, this can help us reveal cases when a species is getting extinct not just due to declining success but due hybridization with another species, which dilutes its genetic integrity [76,77] or when an industry is vanishing solely because it has become obsolete versus when it is evolving into new industries.

Secondly, analysis of emergence dynamics gives us tools to describe entities in research fields where we lack natural intuition of the entity boundaries (e.g. in microbial communities) or foresee a new entity, such as a new scientific field, forming even when it has not yet reached the integrity that would allow intuitively defining it. For example, novel scientific research fields tend to start without a name or rigid boundaries, as vaguely defined collaborations among people from very different backgrounds and proceed to become more coherent programmes with more specialized players and well-defined boundaries [14]. Paralleling this, Bettencourt *et al*. [78] showed that the network of co-authorships unravels and re-structures in the onset of a new scientific field emerging. Lastly, parallel analysis of emergence and success dynamics can be insightful because these aspects of change are intimately linked: our perception of an entity's success depends on the definition of that entity, which in turn depends on the structuring of its features. And then again, the structuring of feature covariation is ultimately guided by the success of each feature combination. In practice, evolutionary change in many fields is or can be modelled like it is done in evolutionary biology—as a joint function of what we call success and emergence dynamics—by modelling patterns resulting from varying feature combinations having varying success, that defines which combinations persist and which features are passed on to new generations [70].

### 3.3. Analysing patterns of rise and decline

#### 3.3.1. Visual illustrations of rise and decline

Patterns of rise and decline can be illustrated in several ways (figure 3). The simplest figure type depicts the hat-shaped curve or spindle-shaped pattern of rise and decline in absolute abundance-based success of an entity (figure 3*a*). An example could be a curve depicting the rise and decline in the numbers of chart-topping songs belonging to a given musical genre. Another common way to illustrate success is through the changes in relative success across a set of entities (figure 3*b*). An example would be a figure depicting how the proportion of top 10 chart songs that belong to particular musical genres changes across time. Here, success is measured by the relative number of units (songs) belonging to an entity (genre), where each unit can only belong to one entity. This plot emphasizes the dynamics of multiple entities competing for the same limited space or resources. Here, again, entities are discrete, but this figure can additionally illustrate how the decline of one entity may be linked to the rise of another taking over.

In addition to success curves, the life history of an entity can be illustrated with plots describing emergence dynamics. For example, if we sample musical features (e.g. harmonic and timbral elements) from a set of songs, we can reconstruct the emergence of musical genres through the increasing frequency at which certain features co-occur across time [3]. This pattern of assembling and disassembling feature sets can be illustrated with an alluvial diagram (figure 3*c*). This plot highlights changes in the modular structure of temporal feature networks, where clusters emerge from *covariation-based* linkage between features, e.g. when two harmonic elements start to occur together (here, in the same songs) more often than they occur separately. Across time, these patterns of co-occurrence describe the gradual build-up of an entity. Alluvial diagrams, visualization tools that highlight changes in modular structure among different network partitions [68], let us notice that these patterns of co-occurrence are born out of decoupling and re-assembling of features from other musical genres.

Few plot types exist that allow us to illustrate compositional emergence and subsequent success dynamics alongside each other. In some cases, it is possible to use a combination of plot types to do this. A clever example are the tree-spindle diagrams (figure 2*d*), especially common in archaeological studies. However, one may also wish to illustrate success and emergence dynamics not separately but as interrelated aspects of change. When an entity's abundance is primarily driven by vertical transmission, and there is direct descendance among units of an entity, success dynamics of an entity may be illustrated alongside some aspects of its emergence using tree plots (figure 3*d*). A tree plot cannot visualize emergence through covariation of features, but it can illustrate the emergence of an entity as a process of descendance: as a branching cluster in the genealogical tree of units. Here, each branching event marks the reproduction of units (e.g. multiplication of bacteria, separation of two lineages) and the entity becomes visible as a branch of the tree. Subsequently, the success of an entity (increased reproduction) becomes visible as a ‘wave’ of denser branching in the tree. These branches will end where the units cease to exist, and across time more and more of them do, resulting in the decline of the lineage as a whole. Any one surviving branch can be the seed of a new descendant entity emerging with another subsequent success wave.

#### 3.3.2. Quantifying success over time

Once we have visualized a pattern of rise and decline of our entities of interest, we can consider different ways to quantify the shape of this pattern in ways that can be meaningfully compared across fields. Firstly, we can measure the *symmetry* of the success curve, that is, the relative steepness of the rise versus the decline. Notably, the shape of the HAT-like patterns can be affected by the rate of evolution (tempo). More or less rapid processes of rise or decline may be signs of different underlying mechanisms. For instance, a slow rise and a rapid decline in abundance of a biological species or an industry may be a result of slow adaptation followed by a catastrophic crash. Conversely, rapid rise and slow decline may result from fast adaptive radiation driven by an innovation or external opportunities (e.g. sudden addition of resources following the disappearance of a competitor), followed by a slow replacement by others (e.g. in the case of prolonged extinction phase termed ‘dead clade walking’ [79]). Rapid rise and prolonged decline have been shown to be common in the histories of biological species [13] as well as in the evolution of scientific fields [14].

Success curves can also be used to measure an entity's *total success* over lifetime, estimated as the area under the success curve. While comparisons of lifetime success are hardly meaningful across different fields, measuring the area under the curve allows us to compare or rank entities within a given system based on their overall success, regardless of the duration of their life span. Ultimately, this enables us to ask questions between fields as well, such as whether the more successful entities (more long lived and/or more abundant) across fields tend to have particular shapes of rise and decline? In other words, is being abundant for a short time or rare for a long time associated with different overall success?

Finally, a key thing to pay attention to in a success curve is its *unimodality*, which can only be tested in retrospect, when the entity terminally disappeared. An absolutely unimodal curve would rise monotonically until it reaches the peak and after the decline starts, there is no going back to the height of the peak. While real success curves are never strictly monotonic in their rise and decline and often include local jitter (small ups and downs), they can be more or less unimodal in terms of prominence of their peak success. Consequently, unimodality typically refers to a single peak viewed at a broad scale. For instance, even though the abundances of animal individuals of a species tend to fluctuate considerably in time, the broad tendency of a species is to have a unimodal peak abundance across their existence [80]. Clearly, the resolution of temporal fluctuations we choose to focus on has great influence over the unimodality of the success trajectory we see. One way to assess this quantitatively is to ‘zoom out’, smoothing the curve with decreasing resolution. By examining unimodality measures across different resolutions of change, we can examine the relative scale of stochastic fluctuations around the hat pattern (trend)—if stochastic changes and trends in an entity's success happen in very different time scales, they are also likely driven by different forces. This type of analysis is common in the classical time-series decomposition (into seasonal components, trends and noise). Paralleling this, the *stability* of a hat pattern can be explored by measuring the relative amplitude of the shorter-scale fluctuations to the large-scale hat pattern or by counting how many times a certain threshold level at the vertical axis of a plot is crossed during the lifetime of an entity.

In addition to the resolution of change, the perceived unimodality of an entity depends on where we set the boundaries of the entity. Can we for instance say that the bloom of cyanobacteria in a single pond comprises a hat pattern with a unimodal peak and irreversible decline, when the descendants of the declining bacteria are more or less predictably bound to bloom again in the next favourable instance? On the one hand we could call these dynamics *cyclic dynamics* of the same entity rising and declining, as it is the same species of bacteria blooming repeatedly. But on the other hand, we could decide that each bloom is the rise and decline of a separate entity, a new lineage within this bacterial species.

Several studies have also highlighted analytical practices that might generate artificially unimodal patterns of rise and decline in success. Pigot *et al*. [81] showed that deterministic-seeming hat-like patterns can be generated by a stochastic model. Hohmann & Jarochowska [82] pointed out that the practice of averaging success trajectories within a rigid time frame to create one summary trajectory (e.g. success of a genus based on trajectories of its species) induces artificial symmetry in the patterns of rise and decline.

#### 3.3.3. Quantifying emergence dynamics

**Figure 4.**
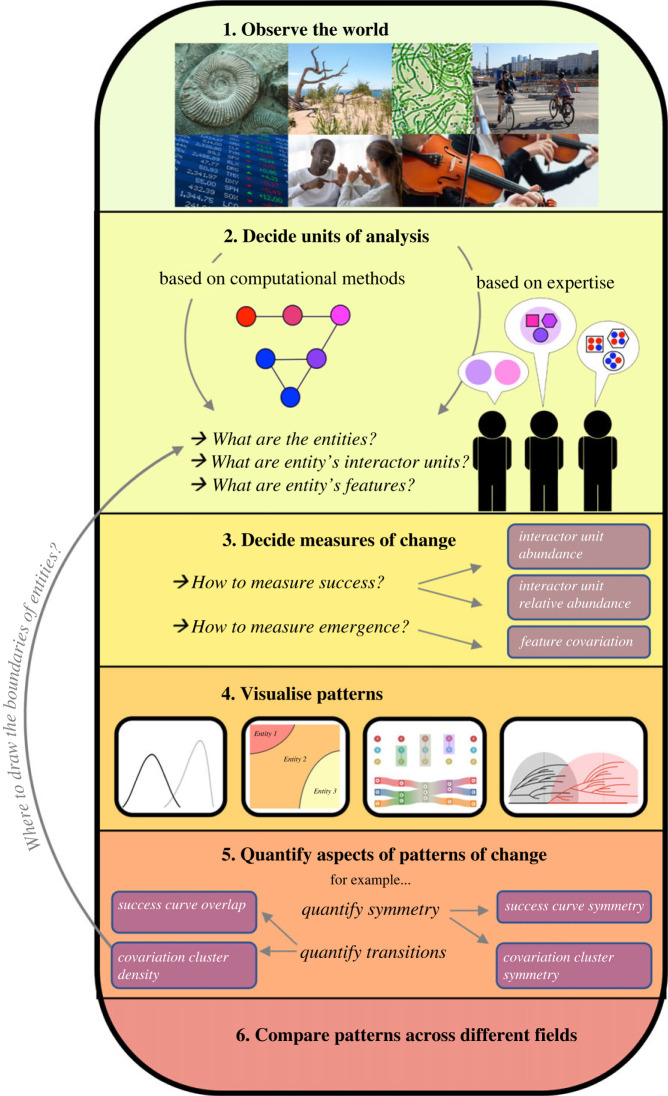
Workflow for interdisciplinary analysis of patterns of change.

When focusing on the dynamics of emergence, we can describe the coming-together and decoupling of an entity through temporal covariation networks of features, or descendance trees of units (figure 3*c*,*d*). These descriptions allow us to use the wide set of analytical tools available for characterizing and comparing structures of networks [83] or trees [84]. Most importantly, these methods help us delineate entities from feature data, aiding the recognition of comparable entities across fields. In addition to identification of entities, interesting aspects of emergence dynamics can be quantified, and compared, just like success curves. For instance, we can assess the descendance relationships of entities through the branching order of a tree, or we may quantify the stability or temporal symmetry of an entity through the topology of the alluvial diagram. Here, paralleling the measures of symmetry in success curves (how fast was the rise and decline of the entity's success), we can also ask how gradual or sudden was the emergence or dissolution of the specific combination of features that comprise the entity. Here, following the semantics of computer science research on concept drift, ‘gradual’ refers to an entity emerging through multiple small steps, for example in a sequence of merging feature sets, while sudden denotes emergence of an entity in one go. By paying attention to the structure of the steps of linkage and decoupling in feature covariation, we can, for example, ask if the winding together and unravelling of an entity follows the same or a different order (figures 3*c* and 4).

### 3.4. Entities in different fields

For each field of inquiry, we identify possible entities to study. For these entities, we identify possible units and features, realistic measures of their success and emergence dynamics and time scales at which these dynamics happen. From here onwards, we will refer to redundant ‘interactor’ units as just ‘units’ of the entity and to functional features as just ‘features’ of the entity. Consequently, when we talk about hat-like patterns in the success of an entity through time, typically we refer to the abundance of units. When we talk about emergence and dissolution of an entity through time, we refer to the arrangement of features. Comparable entities, units and features are summarized in table 1, which also references datasets that can be used to quantify patterns of rise and decline in success or content.

**Table 1.** Summary of some example entities, suggested units and features to measure their success and emergence dynamics and datasets to study these dynamics across fields of inquiry.

research field	evolutionary entities	possible units	possible features (measures of emergence)	measures of success	temporal scale	example datasets
macroevolutionary studies	species, genera and other higher taxa, e.g. evolutionary faunas	individuals of species, species of higher taxa	phenotypic features	entity level: geographical range, occupancy, niche breadth	millennia–aeon	PaleoDB [85]; NOW: database of fossil mammals (2022)
				unit level: abundance (e.g. number of species in a genus)		
linguistics	languages, speech communities	speakers of a language/speaker community	linguistic features (e.g. phonology, morphology, syntax and semantics)	entity level: geographical range; ‘niche breadth’ = number of domains where the language can be used	decades–centuries	WALS [86]; ethnologue [87]; CLICS [88]
				unit level: abundance (e.g. number of speakers of a language)		
industry economics	industries	firms of an industry	routines, technologies or capabilities	entity level: product variation, production volume	years–decades	Thomas Register of American Manufacturers (e.g. [89])
				unit level: abundance (e.g. number of firms within an industry)		
human mobility research	transport modes	users, vehicles or trips within a transportation mode	functional features such as designs and technologies	entity level: geographical range, existing infrastructure	years–decades–centuries	travel times and distances by travel modes in Helsinki [90]
				unit level: abundance (e.g. number of vehicles or trips of a given mode)		
musicology	musical styles/genres	compositions belonging to a style	harmonic and timbral elements	entity level: geographical range, ‘niche breadth’ = context range (diversity of listening contexts)	years–decades	Western classical music [3], popular music [27]
				unit level: absolute or relative abundance (e.g. number of compositions within a genre)		
microbial ecology	microbial strains or higher taxa, microbial genes, microbial community types	individual microbes belonging to a taxon, copies of a microbial gene, microbial communities of a given community type	gene variants across individuals of a taxon, microbial taxa across communities of a community type	entity level: occupancy, niche breadth of a strain, diversity of a microbial community type	hours–millennia	baboon microbiome time series data [91]

#### 3.4.1. Macroevolutionary studies

Macroevolutionary studies focus on analysing the dynamics of higher-level entities in the hierarchical organization of life [92–94], such as the four global mega-assemblages that sequentially dominated oceans over the last 550 Myr in Earth's history [68]. The success of these macroevolutionary entities is estimated from the fossil record through measures such as abundance of units (number of species or higher taxonomic groups) [95], occupancy across measured sites or geographical range [96].

Various hat-shaped success curves have been recognized in the fossil record: distinct patterns of increase to a short-lived peak followed by a decline toward extinction have been documented in the richness, geographical distribution or range of organisms over their duration [39]. To increase robustness success curves are commonly constructed on taxonomic levels above species [97], such as genera or families. Since marine invertebrates are the most common organisms retrieved from the sedimentary fossil record, their occurrence data are widely used to analyse patterns of rise and decline in macroevolutionary research. These hat-like success curves commonly span across time scales of millions of years (figure 2*b*). Concurrently, different aspects of emergence dynamics of higher ranked taxonomic groups are explicitly illustrated by phylogenetic trees, depicting taxa's emergence and disappearance as branching events. Phylogenetic trees can be constructed from genomic [98] and morphological data [99].

#### 3.4.2. Linguistics

Linguistics focuses on the study of spoken and signed languages and language families, language users (speakers/signers), linguistic communities and linguistic features (often grouped into the categories phonology, morphology, syntax and semantics). We use the term ‘language system’ when speaking of language as a sum of its features and ‘language community’ when focusing on language users in space and time. Language systems emerge and disappear across history through patterns of continuous change in content, as measured by composition of linguistic features, as well as rise and decline in their success, as measured by the size of the language community. Traditional historical linguistics compares languages to species: they are inherited, or ‘vertically transmitted’ from parents to descendants, though often with additional horizontal influence from other languages with which they come into contact. As they are passed down generations, they evolve and split into new dialects and languages and the boundary between these is difficult to define [61]. This splitting can be modelled as genealogical trees, analogous to the evolution of biological lineages, and these trees of vertical descendance have been shown to be robust despite realistic levels of horizontal borrowing of elements across languages [100]. Similar to biological species, languages may go extinct if transmission to a new generation fails. For example, the Celtic language Cornish is said to have gone extinct at the end of the 1700s [101] through a language shift to English (there is now a revival movement, but note that revived languages are usually regarded as a new variety, not to be equated with the earlier, pre-extinction variety).

Instead of a language system or community, we can also study a particular speech community as the entity that changes. For example, English is not spoken by a single community but by a number of different dialect communities across the world (e.g. North American English, Sri Lankan English). Paralleling ecological dynamics, languages can undergo patterns of rise or decline within a particular community; they may go extinct in one community while surviving in others. Just like separate lineages of cyanobacteria can bloom (and go extinct) in separate ponds independently, each language community can be thought to possess one unique *lineage* of the language, with its own dynamics of emergence and success. The timeframe of language change depends on the unit of focus. Languages developing into new forms (e.g. Old English into Middle English, or Latin into Old French) is measured in centuries. However, if we focus on features (such as the new second person singular pronoun ‘you’ in figure 2), an innovative linguistic feature may overtake an older feature in the space of a generation.

Importantly, linguistics research on lifespans of languages is in many ways more focused on emergence dynamics than success dynamics. Here, language systems are perceived as emerging through rise, continuation and adaptation of combinations of linguistic features, such as phonemes, lexemes or syntactic patterns. In addition to the transmission and recombination of these feature sets through vertical inheritance, languages can borrow such features from others through horizontal contact, often involving multilingual individuals [102]. Thus, it is possible for features to spread between languages, such that features can survive even if the donor language goes extinct. Examples include features borrowed from Maori into New Zealand (NZ) English [103]. Maori is classified as threatened [104], and if it were to go extinct, some of its features could be said to survive in NZ English as substrate features. In this sense, the borrowing of linguistic features may be compared on a general level to horizontal gene transfer in microbial species, and the co-occurrence patterns of linguistic features can be used to map the emergence and disappearance of languages with emphasis on gradient-like change in their content.

#### 3.4.3. Musicology

In the study of musicology, a common approach is to focus on the historical emergence and success waves of musical styles and genres. For example, in Western classical music, the Baroque period (approx. 1600–1750) was followed by the Classical period (approx. 1750–1800), and further by the Romantic period (approx. 1800–1900). During a given period, many composers created music that contained similar elements, and in retrospect these similarities have been used to categorize the style or genre. For example, during the Classical period, it was common to use the sonata form as a starting point for the structure of the first movement of a musical work. Similarly, power chords played by electric guitars became a common harmonic element in heavy metal.

Across the history of music, the entities that rise and decline are essentially *musical ideas*, such as styles, genres or composition techniques. These can be thought to consist of units, such as compositions/songs or listeners, whose number can be a measure of success of a style over time. Alternatively, when describing the emergence of musical ideas, they can be thought to consist of features such as elements of melody, rhythm and harmony. The co-occurrence patterns of these musical features across songs can be used to trace the emergence and dissolution of a new style [3,27]. Musical styles can evolve more rapidly than species or language systems, in the course of years rather than centuries, and as with many aspects of human culture [105] the speed of their evolution has accelerated in modern times. Composers become successful when they create music that is interesting to listeners and introduce new musical ideas over time. The rise of a musical idea occurs when an interesting combination of musical features has been found and many composers subsequently start using it. Then, the decline of a musical idea can occur when it ceases being interesting through negative frequency-dependent selection [106] or other forces of outdating. For example, while the Baroque style was very popular in its time and known for great masters such as J. S. Bach, no serious composer would create Baroque style music today. The listeners already know the Baroque style and to become popular, modern music is expected to contain new and original combinations of elements.

#### 3.4.4. Industry evolution

In the study of the rise and decline of industries, the focus is on changes in firm numbers and their production volumes. Here, the entity of interest is a given industry, consisting of units such as firms. The success of an industry can be measured for example through production volume, product variation (range) or number of firms (units) within an industry. An industry can also be thought to consist of a combination of features such as routines or capabilities, which can be inherited from one firm to another when a new firm is founded by former employees of a previous firm [107]. These features are essentially the functional units that define an industry: routines refer to ways of doing things [57], whereas capabilities are more complex combinations of resources [108].

New industries emerge through a technological discontinuity that gives rise to a new combination of features, resulting in a new product class. At first, there are few firms entering the industry as there is uncertainty over the viability of the new market, i.e. the demand for the new product class. As technological and market feasibility are achieved, firms enter the emerging industry in increasing numbers. Many of them aim at growth in order to achieve economies of scale. Unit costs of production are usually lower for larger volumes and therefore firms aim at increasing their production volume. As many firms are growing, the capacity of the market is eventually overshot and the majority of firms subsequently exit. A key determinant of survival is the ability to ramp up production to take advantage of economies of scale. As an industry goes through this boom-and-bust process, its composition changes from a large number of small firms to a small number of large firms. Even though in this process the *number of firms* may collapse drastically, the industry reaches its peak *production volume* only after the shakeout where excess firms are pruned away. The time-scale in studying industry evolution ranges from decades to centuries. A typical timeframe from industry emergence to shakeout is 20–30 years in classic examples, such as car manufacturing. More recently, far shorter (less than 5 years) timeframes have been observed, because information diffuses far faster and entrepreneurs are able to learn about new technological opportunities in almost real time [109].

In a young industry, the entering firms are more variable, i.e. introduce different product variants. These can be seen as tests on consumer preferences. Some firms exit because their product fails this test. Other firms adapt to new information by changing their product type. This results in decreasing variety. This change also allows firms to take advantage of economies of scale as the number of variants under production becomes smaller. This is obvious in bulk products, such as pulp and paper [29], but it can also be seen in the video game industry where the game developers focus on an ever-decreasing number of genres [110].

Paralleling the dynamics of novel scientific fields [14], the composition (the combination of features) of an industry tends to be more variable, and boundaries more blurry, early on. As the industry ages, its internal variation decreases and boundaries tighten, resulting in a more well-defined entity. Studies of industries are perhaps a particularly good example of the interplay between dynamics of emergence and success across an entity's lifetime. With the emphasis on the process of testing variations and pruning unsuccessful firms, the overall success of an industry is intimately linked to the process of converging of its content—the trial-and-error-driven emergence of a stable combination of features most fitted to their current environment. This success, however, is transient as environmental change may make the combination of features obsolete.

#### 3.4.5. Human mobility

The study of human mobility is a part of urban sciences that considers patterns of transportation in relation to societal themes, such as inequality [111] or the impact of transport technologies. Mobility research focuses on both short-term dynamics [112] and long-term historic-economic trends [113] in how people get from one place to another. Humans use technologies to move. The evolution of technology has been characterized as an autopoietic ‘combinatorial evolution’, resulting in new reconfigurations of previous technologies in new opportunity niches [114]. The modes of transport (i.e. vehicles, widely understood) thus constitute relevant socio-technical entities to characterize the evolution of human mobility patterns across history (e.g. from Mesopotamian chariots to electric scooters) and in the short term (e.g. scooter dynamics during 1 day).

The success of a given transport mode is often measured in relative scale, for example vehicle stock per area or per capita, or as modal share over trips (e.g. percentage of trips done by car, train, etc.). In addition to relative measures of success, the absolute prevalence of a transport can be measured by its long-term cumulative volumes: produced units, distance travelled by it, energy consumed. Both relative and absolute success can be studied across multiple spatial scales, from city to regional to global.

Historically, each transport mode seems to follow a unimodal pattern of rise and decline in success, with the turn from the peak success coinciding with the rise of a new transportation mode. In this fashion, sailing boats were replaced by steam boats, and these by motorboats. In a similar manner, the use of horses for urban transport peaked around 1900 [54] around the time when car was invented in 1886 (Carl Benz's patent for a ‘gas engine vehicle’). The pattern of rise and decline in automobility (‘peak car’ in the transport literature) has been theorized [55] but also criticized as limited to specific contexts [115].

While transportation modes seem easy to categorize, their change can also be gradual enough for the emergence of a new mode to be a relatively ambiguous process. Consequently, the challenge in studying their patterns of rise and decline is similar to other fields in that the boundaries and definition of an entity are sometimes hard to set, while the historical success dynamics depend on this definition. For instance, should we define all velocipedes as versions of the same entity, from the original 1817 Laufmaschine to geared bicycles to current e-bikes, or do we define these as distinct lineages, each with its own success curve? From this latter perspective, there has been a historic series of unimodal hat patterns of separate entities, but whether this reflects new variants displacing old ones is less clear. For example, while in China traditional bicycles have declined coinciding with the rise of their ‘descendant’ electric bicycles, these success curves are not necessarily describing a ‘transition’ from one to the other [56]. Likewise, the rise of electric and hybrid cars in Norway [116] is not directly related to the abundance of gasoline cars, as the process is not a zero-sum game. Paralleling biological evolution of new species with new ecological niches, different mobility entities may evolve from a previous one, but after this they do not necessarily compete for the same limited resources, because with each new entity some variation appears in their ability to use the space they exist in: compared to bicycles, e-bikes travel further in a given time, and electric scooters are easier to steer and store in urban space.

#### 3.4.6. Microbial ecology

In microbial ecosystems, patterns of rise and decline are frequently observed across entities belonging to various levels of organization: first, within microbial communities we can measure the success of microbial strains*.* Strains are low-level lineages that emerge continuously either by evolving or by spreading from outside the community. The success curve of a microbial strain depicts how they grow and diminish in abundance or occupancy (e.g. number of copies of a strain, number of hosts infected by a strain), a pattern familiar for instance from rise and decline of pathogens during epidemic breaks [117]. Secondly, due to the tendency of bacteria to share genes horizontally, microbial genes can have their own rise-and-decline dynamics that can be independent of the shifts in strain composition. In this way, specific microbial genes such as antimicrobial-resistance genes [118] can emerge, spread, increase in copy numbers, and eventually decline in microbial communities, independent of the taxa of bacteria present. Thirdly, just like in macroevolutionary studies, microbial success dynamics can be traced based on lineages on higher taxonomic levels, such as genera, families or even phyla. Lastly, in some cases these dynamics of microbial genes, strains or higher taxa can feature into the emergence and success dynamics of higher-level entities that can be called *microbial community types.* These entities emerge, exist, dissolve and make way for new types of communities through processes of ecological succession [119,120]. The success dynamics of a community type can be measured through the number meta-communities belonging to a given type (e.g. number of people with a gut microbiome of a given type). The emergence dynamics of a community type can be measured through the co-occurrence patterns among features, which here are microbial taxa. Compared to many other entities, microbial community types provide an example of an entity with a highly unstable composition. Consequently, understanding of emergence dynamics is important for their analysis. As such, the dynamics of emergence and success in microbial community types are perhaps more comparable to dynamics of music genres than, say, biological species.

In microbial ecosystems, the environmental conditions can change rapidly rendering once-useful genetic and phenotypic features obsolete. In such circumstances, it is common that a lineage of a rare microbe may suddenly rise in abundance rapidly, often declining soon after, driven by the sudden emergence of opportunity [121]. Notably, these hat-like patterns in microbiology describe the rise and decline of taxonomic *lineages* just like in macroevolutionary studies, but due to the much finer temporal scale these rising and declining lineages are sometimes harder to recognize as different entities. For example, after an epidemic wave, a new wave may be caused by a strain evolved from the survivors of the previous decline, and this distinct pattern of rise and decline can even be used as a way to delineate the entities from each other, such as in the case of the separately named strains and associated success waves of the coronavirus pandemic.

Dynamics of emergence and success in the microbial world can be rapid or slow depending on the taxonomic level and time scale of interest: when tracing the rise and decline of a new genetic variant or a new strain in a microbial community, these changes can happen in a matter of days [121,122]. On the other hand, considering the dynamics of higher-level microbial taxa (species, genus, family) forces us to map the patterns of rise and decline across larger time scales. For example, some prevalent microbial taxa specialized in living in the guts of animals have risen in their abundance and prevalence in the evolutionary process of phylosymbiosis together with their host species [123].

## 4. Discussion and conclusion

Entities in nature change, or evolve*,* in a continuous, albeit rarely linear, manner. This change can be perceived as the entity rising and declining across its life history. Whether something rises and declines just because it is becoming more or less abundant or because it is gradually changing to another thing is harder to perceive. We highlight two perspectives towards the rise, persistence and decline of an entity: one reflects the dynamics of success, the other reflects the dynamics of composition. In the former case, the definition of the entity stays the same throughout its existence, only its abundance or other measure of success rises, peaks and declines until the entity disappears due loss of success. In the latter case, the rise and decline concerns the emergence and decoupling of a set of characterizing features that define the entity. Dynamics of success and emergence are not independent of each other. Firstly, lifetime success of an entity is a measure only applicable to entities with discrete boundaries, and setting these boundaries often depends on our understanding on how they emerged. Secondly, entities emerge as sets of features guided by the success of these combinations. For example, at each generation, recombinations of gene variants are what natural selection acts on to guide evolution of biological species [70].

Comparable data to study patterns of rise and decline exist among many fields of research. Across our six fields of inquiry (macroevolutionary studies, microbial ecology, linguistics, study of industry evolution within economics, musicology, human mobility studies), we identified comparable entities, units and features and suggested measures for their success and emergence dynamics based on these. The next step would be to quantify these dynamics across multiple field-specific datasets to examine what tendencies of success and emergence are universal across contexts, and what may be the important variations between different fields. Field-specific differences in dynamic profiles of entities are likely to exist for a number of reasons. Firstly, the boundaries of entities in some fields may be more stable than others, resulting in the change in an entity's content being more or less gradual. For instance, biological species, despite their history of descendance from each other, are considered relatively stable entities [122]. Biological species consist of populations that show individual and temporal variation in content (e.g. gene variant frequencies) but this variation is lesser during their stable period of existence as a species compared to the rapid periods of evolution that happen when either selective forces or reproductive isolation between populations accelerates their divergence from their ancestral population. Delineating entities in more gradually changing feature sets is less obvious, and this is why computational methods for measuring emergence dynamics may become more important in characterizing entities such as musical genres or microbial community types.

Another reason why patterns of rise and decline may differ between fields is that the dynamics of different entities happen at very different scales. There is no single optimum scale to describe the evolution of entities but there may be multiple scales that capture dynamics at different levels in the complex hierarchical structuring of systems. For instance, dynamics of life can be viewed as anything from fluctuations in gene variant distributions to dynamics of cultures, species abundances and all the way to the emergence and decline of taxonomic assemblages. Despite differences in scales research could benefit from exploring the stability of hat-like patterns or patterns of feature change across scales. While similar universal patterns of change are perhaps expected regardless of scale, the ratio of change to the observational period in the data influences the dynamics we are able to perceive. Donker's theorem suggests that stochastic dynamics tend to look different (and spurious deterministic-looking patterns may be more common) when the scale of their variation is larger relative to the size of the observed space, i.e. when the resolution is coarse [123].

A more nuanced understanding of patterns of rise and decline can lead to new means to notice, anticipate and manage change, and new tools to analyse temporal data across a variety of disciplines. This can help foresee dramatic events, such as predicting catastrophic crashes in cultural, ecological, economic or climate systems, which have been an intriguing programme for interdisciplinary systems research. For example, Holling's adaptive life cycle theory attempts to describe fates of socio-ecological entities (e.g. ecosystems, infrastructures, cultures) as more or less deterministic cycles where the decline phase of an entity is a predictable consequence of the accumulating rigidity of its structure in the previous state [16]. Our framework treats entity dynamics non-deterministically—for instance, rather than predicting when and why an entity is going to start declining, it provides tools to characterize the onset of decline, even in retrospect, which eventually ends in the extinction of the entity.

With a more comprehensive understanding of emergence and success dynamics as well as their interplay, we can start building tools for not only anticipating change in success (e.g. catching the onset of decline before catastrophic crashes) but also recognizing when a new entity of importance is in formation or when a recognized entity is losing integrity due to becoming something else. For example, following Singh *et al*. [14] novel scientific fields are hardly perceived as fields in their early phases of formation. This early phase, however, may be the most influential time for the future content and success of the field. With tools to analyse emergence dynamics, we could recognize the moments when something influential is emerging, even before it lacks the integrity to bring it a name and identity as such. Furthermore, in addition to phenomena, conceptualization of emergence dynamics can open up an avenue of research on patterns that we would hardly perceive otherwise. For example, by recognizing features as elements that both define an entity and yet can spread from one entity to another, we may study the history of a feature, across multiple entities it may belong to during its lifetime. Some features, such as gene variants or musical harmonies may ‘surf the success wave’ of one entity, only to hop on to the next as the former entity declines. This way the same gene variant can have a history spanning across multitudes of already extinct species, just like a word can outlast a language or a harmony coined up in Baroque can find its way to modern styles of music.

**Figure 5.**
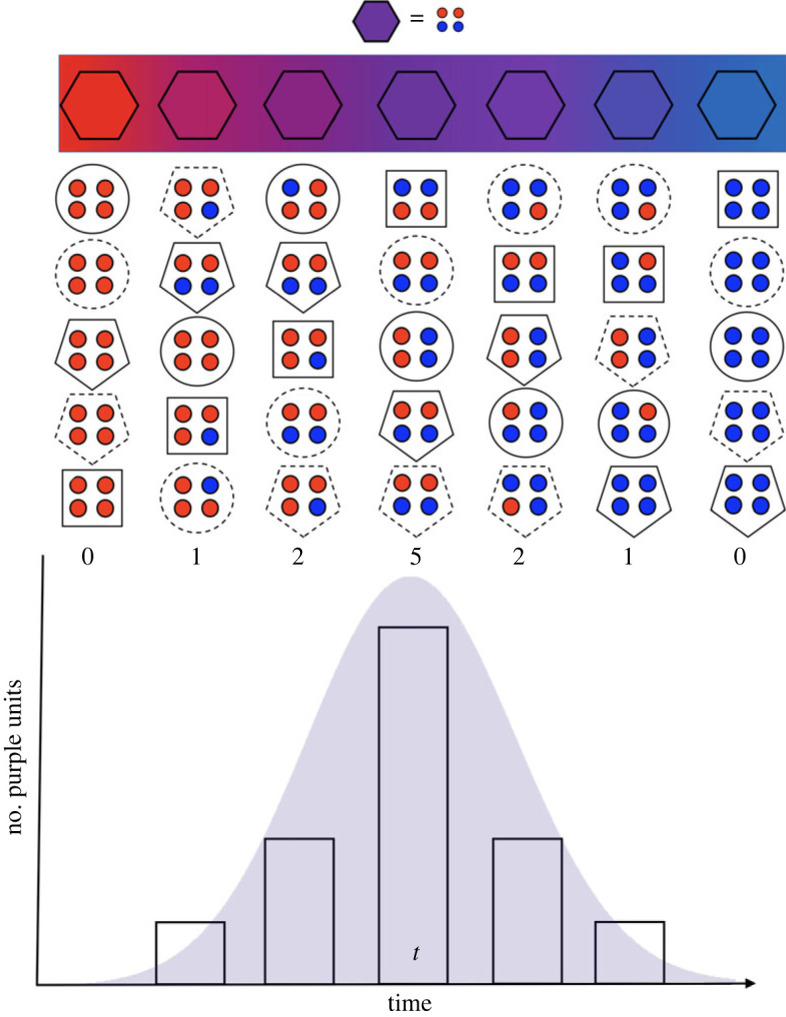
Illustration of how gradual change in an entity's feature content (upper panel colour) can be perceived as rise and decline of a discrete entity. Let us define our entity using the distribution of features (red and blue dots) across variable units (shapes) at a single time point *t*. Since all of our units have 50% blue features and 50% red features, we can say that our entity's identity is purple by definition. In a gradually changing population of units, the units that satisfy the criteria of belonging to the entity (being purple) tend to increase towards time point *t* and decrease after time point *t*. This way we can perceive a gradual, ‘gradient-like’ change from blue entity to red entity as the rise and decline of purple.

Considering dynamics of emergence and success in parallel is worthwhile also because it can help us to critically evaluate the definitions of the entities we focus on in our analyses. When we categorize entities (e.g. genres, industries, species or languages) to summarize variation that naturally exists in a more or less continuous state of change, we need to be cautious of over-simplification [124]. This may be more relevant now than ever since we are living an unprecedented era of instability across natural, cultural and societal systems, but this does not mean that we should abandon the idea of a stable system in our quest to understand the dynamics of the world [1]. The success curve of an entity is telling the story of rise and decline only in relation to a static definition of an entity, and measuring the success of such a static definition makes the more sense the more stable the entity is in its content. If we choose an entity with a relatively gradual mode of change, and define it based on one static state, a symmetrical hat pattern may form around the time point of the state we use to define the entity (figure 5). As an example, if we define a language as an exhaustive set of words and grammar present at a given time point, the history before this point will seem like the rise of this particular feature set and the subsequent gradual change in the language's content will seem like a decline in the abundance of this particular feature set. However, most entities do not change at a constant rate but exhibit tipping point behaviour (also known as ‘threshold behaviour’), meaning they exist in relatively stable states (or their definitions rest on a relatively small number of stable key features) and change between these through more rapid shifts.

In practice, research on patterns of rise and decline in nature including society rely on discrete entities largely defined by qualitative approaches with deep historical roots (e.g. languages delimited on the basis of geopolitical history). Importantly, the dynamics of life we are able to perceive depend on these definitions of the relevant entities. How these definitions come to be is a topic worth exploration in humanities as well as sciences. Specifically, when our entity of interest is defined based on intuition alone, we are always going to make better-informed delineations in fields where we have better intuition. For example, we can intuitively categorize music variation as ‘genres’ in a way that corresponds remarkably well to quantitatively recognizable co-variation clusters of musical features [3,27]. Similar intuition may not be possible for more cryptic entities, such as microbial community types. These entities may not be intuitively categorizable, but can nevertheless be influential and relatively stable agents of life, just like semantically more familiar entities, such as ‘industries’, ‘species’, ‘dialects’ or ‘genres’. To overcome biases in intuition accuracy between fields of study, we can use quantitative methods, such as clustering of feature networks, to define our entities from some feature variation data. Of course, even when using quantitative approaches, we will always be limited by our human biases, data and chosen definitions for the units and features of entities.

Our ability to intuitively perceive coherent entities varies not only between fields of science, but also between cultures and lifestyles. For instance, modernization and urbanization in humans have been linked with enhanced intuition in recognizing variation in brands [125] and decreasing ability to recognize variation in local ecosystems [126]. In addition to modernization, the cultural view-points that can influence our perception of entity boundaries and dynamics likely vary between human populations. Notably, most of the research on rise and decline of entities comes from researchers with a Western background. This means that while the patterns of rise and decline have been similarly recognized by people from various disciplines and centuries apart, many of them still come from the same narrative tradition, that of Herodotus. Whether studies of system change would have different emphasis points, such as patterns of more continuous change and less discrete entities, when studied within the influence of other narrative traditions is a topic worthy of further consideration. Overall, the diversity of perspectives on change we possess as humankind is not only a matter of biases but a tool kit for understanding complex phenomena. Pooling human knowledge on system change can also mean using the rich set of perspectives, metaphors and mathematical ideas on patterns of change across fields of research as well as across the gradient of human thought cultures.

## Data Availability

Electronic supplementary material is available online [127].
